# The Perception of Physical Activity and Sports Professionals’ Competence in Working With Individuals With Disabilities in Spain

**DOI:** 10.3389/fpsyg.2021.735325

**Published:** 2021-11-04

**Authors:** María Gutiérrez-Conejo, María-Dolores González-Rivera, Antonio Campos-Izquierdo

**Affiliations:** ^1^Department of Biomedical Sciences, Faculty of Medicine and Health Sciences, University of Alcalá, Madrid, Spain; ^2^Department of Social Sciences of Physical Activity, Sports and Leisure, Faculty of Physical Activity and Sport Sciences, Technical University of Madrid, Madrid, Spain

**Keywords:** competence, professional, physical activity and sport, disability, professional quality

## Abstract

The importance of professional competence lies in the effective application of job-oriented knowledge and skills which guarantee one’s successful adaptation to the work. This study analyzes the perception of the importance of physical activity and sports (PAS) professionals’ competence in working with individuals with disabilities in Spain. As a descriptive quantitative study, face-to-face interviews were conducted through a survey to extract the data. The sample consisted of 214 PAS professionals working with people with disabilities. According to the results, the analyzed constituents of professional competence are important for adequate performance (>65%), with the exception of competences of leadership and use of new technologies (<50%). It was also found that the perceived importance of each element of professional competence varies according to age, experience and training. Based on the obtained results, the degree of importance of each constituent of professional competence and its implication for the access of people with disabilities to high-quality physical activity and sports services was determined.

## Introduction

The ratification of the convention on the rights of people with disabilities in 2007 commits the Spanish State to designing, developing and implementing policies, laws, and measures accordingly (Leardy-Antolín et al., 2018). In order to guarantee proper assistance and service, one of the measure is to ensure that professionals, who work with the disabled individuals, are specially trained (Boletín Oficial del Estado, 2008).

The main objective of university education in Spain is to equip students with the necessary employability skills and competence along with personal and professional development (Royal Decree, 2007 which establishes the organization of Official University Education). There is a fundamental connection between the initial training and the acquisition of professional skills (Campos-Izquierdo and Martín-Acero, 2016). The initial training provides one with the fitting knowledge, skills, attitudes and procedures in a complementary way (Pereda and Berrocal, 1999; Campos-Izquierdo, 2010). In the next step, one will be able to demonstrate the acquired competence in knowledge and personal, social and methodological skills in educational or professional contexts (European Parliament, 2008).

The European Council considers competence as the set of socio-affective behaviors and cognitive, psychological, sensory and motor skills that allow an activity or task to be adequately performed (Commission of the European Communities, 2005). Its importance lies in the application of the knowledge, procedures and skills to the job, guaranteeing the adequacy of the professional performing it (Aldana-Becerra and Ruiz, 2010; Agencia Nacional de Evaluación de la Calidad y Acreditación [ANECA], 2013). Therefore, competence constitutes a basic element of professional development (Aldana-Becerra and Ruiz, 2010; Campos-Izquierdo and Martín-Acero, 2016) and is fundamental to competitiveness, individual development, social cohesion and employment (European Parliament, 2008).

The professional competence model consists of three levels, ranging from the most universal to the most concrete. There are first the basics: a combination of skills, knowledge and attitudes adapted to different contexts (Commission of the European Communities, 2005). Next there is the layer of generic competence, incorporating knowledge, abilities and skills which can be extrapolated to any job position (Pazo and Tejada, 2012). The generic competence can be classified into instrumental, interpersonal and systemic competence (ProyectoTunning, 2003).

Lastly there is the layer of the set of specific competence, which forms the basis of professional practice and is directly linked to the job position (Pazo and Tejada, 2012). The competences related to the professional performance and labor market of the PAS are stated in the Resolution of September 18, 2018, which verifies the Bachelor’s degree in Physical Activity and Sports Sciences (PASC), and in the work by Campos-Izquierdo and Martín-Acero (2016):

1. *Educational intervention.*
2. *Prevention, adaptation and improvement of physical-sports performance and health through physical condition and physical exercise.*
3. *Promotion of healthy and autonomous habits through PAS.*
4. *Intervention through physical exercises.*
5. *Planning, evaluation and direction/organization of resources and of PAS.*
6. *Scientific methods and evidence in practice.*
7. *Performance, deontology and professional practice in the context of the interventions.*

The PASC graduates must have cross-sectional cognitive training, effective multidirectional skills and competences that help them adapt to all work situations with a guaranteed level of professional performance (Ballesteros et al., 1994). The ability to integrate these competences is the main characteristic of the PASC graduates and the one that differentiates them from those of other disciplines (Campos-Izquierdo, 2010).

With the growing number of people with disabilities who participate in PAS, the lack of training and professionalization of specialists in this area has become evident (Barnet et al., 2015; Reina et al., 2019). This has lowered the competence self-perception during work performance (Nascimento et al., 2007; Díaz-Cueto, 2009; Sanz and Reina, 2012; Castro et al., 2018).

The lack of specialized training programs for PAS professionals who work with individuals with disabilities in Spain (Barnet et al., 2015) indicates the importance of establishing a proper, consensual competence profile (Jiménez and Hernández-Álvarez, 2013) which along with providing standardized PAS training, is aligned with the demands (Nichols et al., 2019) and ensures the access of people with disabilities to quality PAS services.

The Resolution of September 18, 2018 calls for PAS professionals to be competent at the following: learning adapted and inclusive physical activity; designing, didactic intervention in and evaluation of adapted physical education and physical exercise by taking diversity into account, and integrating knowledge on PAS and disability issues. In addition, the training and competence standards that guarantee the adequacy of the PAS professionals working with people with disabilities were collected in the studies by Hutzler and Sherrill (2007); Ferreira and Morgulec-Adamowicz (2011), and Klavina and Kudlácek (2011), taking the management, planning, monitoring and adaptation of sport as reference areas of competence.

In this context, the main objective of the present study is to determine the level of importance that the PAS professionals who work with people with disabilities in Spain attach to general competences. The obtained indicator can be used as a frame of reference for specialized PAS training. The professional who acquires these competences, can provide quality PAS for the individuals with disabilities. The specific objectives of the study are:

- To analyze the level of importance conferred on professional competence by the PAS professionals who work with people with disabilities in Spain.

- To examine the level of importance of professional competence perceived by the PAS professionals working with people with disabilities in Spain based on gender, training, age and experience.

### Hypothesis Development

The search for unanimity in the professional competences necessary for an adequate PAS professional performance has been a line of research. Campos-Izquierdo and Martín-Acero (2016) consider the main competences chosen by the PASC to be planning, designing, developing and evaluating physical condition and physical exercises. In the study carried out by Romero et al. (2011), teamwork, innovation and creativity are viewed as the key competences. Other studies suggest planning, ethical behavior, decision-making and problem solving (Batista et al., 2008; Pazo and Tejada, 2012). Based on these references, the present study addresses this question: Do all the competencies guarantee an adequate level of PAS for people with disabilities. The following hypothesis is proposed:

H1 The major professional competencies in PAS performance include planning, evaluation, professional ethics, decision-making and problem solving.

The research on the PAS for people with disabilities focuses on the analysis of training and competence self-perception during professional performance (Kelly and Gansneder, 1998; Díaz-Cueto, 2009; Crawford, 2011; Klavina and Kudlácek, 2011; Jiménez and Hernández-Álvarez, 2013; Castro et al., 2018). The results show the lack of proper specialized training of PAS professionals working with individuals with disabilities and low levels of competence perception in professional performance. In other PAS occupations, the perception of competencies is related to professional training and experience (Boned et al., 2006; Valle et al., 2015; Ojeda-Nahuelcura et al., 2022). Moreover, the factors of age and gender seem to influence the perception of competencies, although the differences are not significant (Valle et al., 2015).

From these studies, it can also be inferred that the perception and choice of competence by professionals can be a key tool in designing specialized PAS training programs (Cunha et al., 2010). To the best of the authors’ knowledge, however, no studies have thus far analyzed the perceived importance of the PAS professionals’ competence in working with people with disabilities. Do gender, age and experience influence the way one perceives professional competencies. To analyze the possible differences, the following hypotheses were established.

H2 The perception of professional competencies is similar between women and men.
H3 The perception of competencies varies between the youngest and the oldest professionals.
H4 Highly experienced professionals have a different perception of the skills, compared to those with less experience.
H5 The perception of competencies varies between professionals with a university education and those with lower levels of education.

## Materials and Methods

A descriptive and quantitative methodology was used to objectively recognize the characteristics of the professionals involved in PAS for individuals with disabilities in Spain (Alvira-Martín, 2004; Lussier, 2009). The sectional survey was used as a procedure (García-Ferrando et al., 2007), carried out through face-to-face questionnaire-based interviews. The interviewers met with the professionals and verbally asked them the questions from the questionnaire and wrote down the answers. This procedure of the data collection provided the participants an opportunity to ask any questions that arose with regards to the questionnaire or the study in general. The interviews were conducted by 19 interviewers who were primarily PASC graduates, and had attended a training seminar led by the main researcher.

This research has received the favorable report of the Ethics Committee of the Polytechnic University of Madrid.

### Sample

The sample consisted of 214 participants, 135 men (63%) and 79 women (37%) with an age range of 16–70 years old involved in PAS for people with disabilities in Spain. The age ranges are 16–29 (35%), 30–44 (48%), and 45–70 (17%) (Gutiérrez and Campos-Izquierdo, 2019). Due to the large size of the population, we have worked with a confidence interval of 95.5%, a population variance *p* = *q* = 50% and a margin of error of ± 2% (Campos-Izquierdo, 2016).

The allocation of the sample is proportional to the distribution of people in the autonomous communities, provinces and municipalities of various sizes (<10.00; 10.001–50.00; 50.001–100.000; 100.001–500.00; > de 500.000 residents). A probabilistic multistage cluster sampling was used and the sample was stratified in the first phase (Cea-D’Ancona, 2001; Perelló-Oliver, 2010).

To select the sample, probabilistic multistage sampling was used. Several phases were stratified: regions, provinces, municipalities, sport facilities and finally the professionals to be interviewed. Random sampling was applied at each phase. Once the interviewers were in the sport facility, they identified the subject to be interviewed and randomly invited them to participate in the study.

This study is part of the Fundamental Research Project of R + D + i DEP2009-12828, with a total sample size of 2.500 people who are involved in the provision of PAS in Spain.

### Instrument

The standardized interviews were based on the questionnaire “PROAFIDE” (Campos-Izquierdo, 2011), which is made up of 57 closed and factual short answer and multiple-choice questions that collect information on the five dimensions: sociodemographic characteristics, PAS job functions, professional performance, job characteristics and training characteristics.

Items related to the objectives of this study were selected from the questionnaire “PROAFIDE.”

The questions cover 10 items related to professional competencies. During the interview, the participants attached importance to each of the 10 professional competencies based on a Likert scale: 0 = “not important,” 1 = “moderately important,” and 2 = “very important.”

These items associated to each competence were studied in relation to four closed questions related to: gender, age, work experience and level of studies. By doing so, data related to professional performance, sociodemographic and training characteristics of each PAS professional were collected.

### Data Analysis

Descriptive statistics and univariate analysis were used to identify the degree of importance of each competence in percentages (%), means (M) and standard deviation (SD). Bivariate analysis was employed to establish the relationship between education, age, gender, experience and the participants’ perception of their professional competencies.

The association between the outcome variables (gender, experience and perception of competencies) was analyzed using *t-*Student’s test. For the outcome variables (age, education, and perception of competencies), a one-way ANOVA test with *post hoc* DSM was performed. The Phi correlation coefficient, Pearson’s chi-square value and its significance were also calculated. The level of statistical significance was set at *p* < 0.05.

The data has been tabulated and computerized using the SPSS software package for Windows (V19.0).

## Results

The results show that the PAS professionals who work with people with disabilities in Spain consider that all professional competences are very important to proper professional performance (>65%), with the exception of leadership and the use of new technologies competences. The competencies which were considered as very important and, therefore, contributing to an adequate professional performance are communication (77%, *M* = 1.76, SD = 0.45), decision-making (74%, *M* = 1.74, SD = 0.44), and problem solving (73%, *M* = 1.71, SD = 0.50) (Table 1).

**TABLE 1:** Levels of importance given to professional competences by the PAS staff working with people with disabilities in Spain.

	0	1	2	M	SD
Problem solving	2%	25%	73%	1.71	0.50
Communication competence	1%	22%	77%	1.76	0.45
Leadership	17%	38%	45%	1.30	0.71
Professional quality	0%	32%	68%	1.67	0.48
Autonomy and initiative	2%	31%	67%	1.65	0.50
Adaptation to change	2%	28%	70%	1.67	0.52
Use of new technologies	17%	38%	45%	1.28	0.74
Planning, organization	2%	27%	71%	1.69	0.50
Decision-making	4%	22%	74%	1.74	0.44
Professional ethics and deontology	1%	28%	71%	1.71	0.47

According to the results, men and women attach similar levels of importance to professional competences, regarding communication competence as very important, with a percentage of 78 and 75% for men and women, respectively. For men, professional decision-making (77%) and problem solving (76%) are also of high importance. Women view planning, organizing, professional ethics and deontology as crucial competences (71%). The professional competences which were perceived less important and, therefore, less effective in specialized PAS include the use of new technologies (men 16% and women 19%) and leadership (men 14% and women 16%).

The responses of the different age groups show that 78% of the participants between 16–29 and 76% of the participants between 30 and 44 years old perceive communication competence as very important, while the participants more than 45 years of age see the professional ethics and deontology (81%) as the main competence. It is noteworthy at for the participants more than 30 years of age, planning, organization and decision-making competences are of significance importance.

Considering professional experience as a factor, the analysis of the data indicates that the less experienced participants put the main weight on the competences of communication (80%) and problem solving (76%). On the other hand, the more experienced ones perceive decision-making (77%) and professional ethics and deontology (78%) the major competences.

The results also show that the importance attached to professional competences varies according to the educational level. For the participants with primary or lower levels of education, communication (91%) plays a major role, followed by the competence of decision-making (78%). The professionally trained participants (81%) view problem solving as the most important competence, with communication coming in second. The data also indicates that for the participants with university degrees ethical and deontological behavior (75%) and decision-making (75%) constitute the key competences, followed by professional planning and organization competences (72%). According to Table 2, there are significant relationships between age and professional ethics and deontology competencies (*p* < 0.01). This is also the case for the training and problem-solving competencies (*p* < 0.01), leadership competence (*p* < 0.01), competence in the use of new technologies (*p* < 0.05) and adaptation to change (*p* < 0.01).

**TABLE 2:** Levels of importance given to professional competences by the PAS staff working with people with disabilities in Spain based on gender, age, experience and training.

		Problem solving			Communication			Leadership			Professional quality			Autonomy and initiative		
						
		0	1	2	0	1	2	0	1	2	0	1	2	0	1	2
Gender	Men%	2	22	76	0	22	78	14	40	46	0	30	70	0	36	64
	Women%	3	29	68	2	23	75	16	41	43	1	34	65	3	29	68
Age**	16–29%	1	24	75	0	23	78	18	41	41	0	33	67	3	28	69
	30–44%	3	23	74	2	22	76	14	41	45	1	34	65	3	28	69
	45–70%	3	30	67	0	22	78	14	36	50	0	22	78	0	28	72
Experience	<10 years experience%	4	20	76	0	20	80	20	36	44	1	33	66	3	27	70
	>10 years experience%	1	29	70	2	25	73	10	44	46	0	30	70	2	29	69
Training*	University degrees%	1	28	71	1	25	74	9	49	42	1	30	69	1	32	67
	Vocational training%	2	17	81	3	28	69	31	31	38	0	45	55	2	41	57
	Primary level%	7	21	72	0	9	91	19	24	57	0	24	76	0	29	71

		**Adaptation to change**			**Use of new technologies**			**Planning organization**			**Decision making**			**Professional ethics and deontology**		
						
		0	1	2	0	1	2	0	1	2	0	1	2	0	1	2

Gender	Men%	2	28	70	16	39	45	1	29	70	4	19	77	1	28	71
	Women%	2	28	70	19	37	44	4	25	71	3	29	68	0	29	71
Age**	16–29%	3	28	69	27	41	32	3	33	64	1	33	66	1	36	63
	30–44%	3	28	69	12	37	51	2	25	73	7	18	75	0	26	74
	45–70%	0	28	72	15	31	54	0	22	78	0	22	78	0	19	81
Experience	<10 years experience%	3	27	70	22	38	40	3	30	67	6	22	72	0	35	65
	>10 years experience%	2	29	69	13	37	50	1	24	75	1	22	77	1	21	78
Training*	University degrees%	1	30	69	13	43	44	2	26	72	3	22	75	0	25	75
	Vocational training%	7	26	67	24	38	38	2	36	62	5	31	64	2	36	62
	Primary level%	2	22	76	24	21	55	2	24	74	5	17	78	0	33	67

(p < 0.01**) (p < 0.05*).

The *t*-test analysis for equality of means shows that there is no significant relationship between gender and the perception of professional competencies (Table 3).

**TABLE 3:** The comparison of means between men and women on the perception of professional competencies.

	M men	M women	SD men	SD women	*t*	df
Problem solving	1.73	1.66	0.49	0.52	0.613	212
Communication competence	1.78	1.72	0.42	0.50	1.125	212
Leadership	1.32	1.27	0.71	0.72	0.648	212
Professional quality	1.70	1.63	0.46	0.51	0.875	212
Autonomy and initiative	1.65	1.61	0.48	0.53	−0.485	212
Adaptation to change	1.67	1.67	0.51	0.52	−0.315	212
Use of new technologies	1.29	1.25	0.73	0.75	−0.316	212
Planning, organization	1.70	1.67	0.48	0.54	0.563	212
Decision-making	1.77	1.68	0.42	0.47	0.898	212
Professional ethics and deontology	1.70	1.71	0.47	0.45	−0.265	212

According to the *t-*test results of equality of means presented in Table 4, there is a significant relationship between professional experience and the competence of ethics and deontology (*t* = −2.58; *p* < 0.01). Furthermore, the results show that the more experienced people perceived with higher importance the competence of professional ethics and deontology (*M* = 1.78, SD = 0.41), in comparison with the less experienced professionals (*M* = 1.63, SD = 0.50).

**TABLE 4:** The comparison of means of the perception of professional competences according to experience (<10 years and >10 years).

	M < 10 years	M > 10 years	SD < 10 years	SD > 10 years	*t*	df
Problem solving	1.72	1.69	0.52	0.48	0.45	212
Communication competence	1.80	1.71	0.40	0.50	1.58	212
Leadership	1.24	1.36	0.76	0.65	−1.25	212
Professional quality	1.65	1.70	0.49	0.46	−1.11	212
Autonomy and initiative	1.66	1.55	0.49	0.54	0.33	212
Adaptation to change	1.68	1.67	0.52	0.51	0.03	212
Use of new technologies	1.19	1.37	0.76	0.70	−1.54	212
Planning, organization	1.64	1.74	0.53	0.46	−0.82	212
Decision-making	1.71	1.76	0.45	0.42	−1.43	212
Professional ethics and deontology	1.63	1.78	0.50	0.41	−2.58**	212

p < 0.01**.

The *post hoc* comparisons show a significant relationship in the perception of professional excellence competence between people with age ranges between 16–29 and 30–44 (*p* < 0.05) (Table 5).

**TABLE 5:** The comparison of means of the perception of professional competencies according to age.

	M 16–29	M 30–44	M 45–70	SD 16–29	SD 30–44	SD 45–70	*F*	df
Problem solving	1.73	1.71	1.64	0.47	0.51	0.54	0.61	2
Communication competence	1.77	1.74	1.78	0.42	0.48	0.42	0.58	2
Leadership	1.24	1.32	1.36	0.73	0.70	0.71	0.54	2
Professional quality	1.67*	1.64*	1.78	0.47	0.50	0.42	0.19	2
Autonomy and initiative	1.75	1.66	1.64	0.43	0.49	0.50	1.98	2
Adaptation to change	1.67	1.66	1.72	0.52	0.53	0.45	0.07	2
Use of new technologies	1.05	1.40	1.39	0.76	0.69	0.72	0.04	2
Planning, organization	1.61	1.71	1.78	0.54	0.50	0.42	2.13	2
Decision-making	1.71	1.75	1.78	0.46	0.43	0.42	0.50	2
Professional ethics and deontology	1.61	1.74	1.81	0.51	0.44	0.40	2.66	2

p < 0.05*.

The results of the ANOVA test show a significant relationship between academic training and the perception of professional communication competence (*F* = 3.65, *p* < 0.02). *Post hoc* comparisons indicate a significant relationship between the perception of the competence of professional ethics and deontology and university training and vocational program (*p* < 0.05). The perception of communication competence and vocational program and secondary school training (*p* < 0.00), as well as leadership competence and university training and vocational program (*p* < 0.05) have also a significant relationship (Table 6).

**TABLE 6:** Comparison of means of the perception of professional competencies according to initial training.

	M university degrees	M vocational training	M primary level	SD university degrees	SD vocational training	SD primary level	*F*	df
Problem solving	1.71	1.79	1.63	0.47	0.46	0.61	1.11	2
Communication	1.74	1.67	1.91	0.46	0.52	0.29	3.65*	2
Leadership	1.34	1.07	1.40	0.63	0.83	0.78	1.90	2
Professional quality	1.68	1.55	1.77	0.48	0.50	0.42	2.33	2
Autonomy and initiative	1.72	1.65	1.61	0.45	0.48	0.53	1.52	2
Adaptation to change	1.68	1.60	1.72	0.48	0.62	0.50	0.78	2
Use of new technologies	1.32	1.14	1.28	0.68	0.77	0.84	0.55	2
Planning, organization	1.71	1.60	1.72	0.49	0.54	0.50	0.68	2
Decision-making	1.75	1.64	1.79	0.43	0.48	0.41	1.05	2
Professional ethics and deontology	1.75	1.60	1.67	0.43	0.54	0.74	1.85	2

*p < 0.05^∗^.*

## Discussion

Generally, the PAS professionals who work with the individuals with disabilities in Spain consider all professional competences to a greater or lesser extent to be important for ensuring quality of their professional practice (Campos-Izquierdo and Martín-Acero, 2016).

The level of importance given specifically to decision-making, communication, and problem-solving competences indicates their substantial contribution to an adequate professional performance (Corominas et al., 2006; Ferreira and Morgulec-Adamowicz, 2011). Therefore, the acquisition of these professional competences guarantees the adaptation to the job and provides the access of people with disabilities to quality PAS.

It can be seen that both men and women similarly view organization and planning, adaptation to change and professional ethics and deontology. The studies by Romero et al. (2011) and Valle et al. (2015) show insignificant differences between men and women in the perception of leadership, relationship and organization and planning competencies.

Based on the analysis of the perception of competencies according to age, the results show differences between the age ranges 16–29 and 30–44. These differences are similar to those observed in the study by Valle et al. (2015). In relation to experience, significant differences were observed in the perception of professional competence in ethics and deontology. Experience as a determining factor in the perception of competencies was also observed in the study by Cunha et al. (2010) and Valle et al. (2015).

The competence standards require that the PAS professionals working with individuals with disabilities achieve these professional competencies (Ferreira and Morgulec-Adamowicz, 2011).

It is also observed that a professional’s level of education affects the importance he/she attaches to particular competences. The participants with a university degree emphasize the importance of the competences of decision-making, ethics and professional deontology and communication, which are partly different from those selected by professionals with lower levels of education. Similar results have been reported by Boned et al. (2006). Other studies, too, regard the planning competence to be of great importance in professional performance (Batista et al., 2008; Cunha et al., 2010; Lytle et al., 2010; Romero et al., 2011). Agencia Nacional de Evaluación de la Calidad y Acreditación [ANECA], 2006) underscored the importance of the competences of organizational and planning capacity, decision-making and professional quality for the PAS professors and professionals.

The competences of leadership and the use of new technologies were the least valued by the PAS professionals surveyed. In the case of the use of new technologies, the same holds true for the physical education teaching (Soriano and Delgado-Noguera, 2011). Despite the recent increase in the use of new technologies in the field of PAS, it is still limited (Tahara and Darido, 2016) by the level of teachers’ technical knowledge and safety concerns (Suárez et al., 2010). It is important to note that the use of new technologies is not among the most valued professional competences despite its flexibile capability to adapt to the needs and interests of different groups, and thus contributing to the inclusion of people with disabilities (Sánchez-López et al., 2014).

## Conclusion

In this study, the professional competences considered most important are communication, decision-making and problem solving, followed by the competences of planning and organization, professional ethics and deontology and adaptation to change. This perception drawn from one’s professional experience indicates that the adequate acquisition of competences guarantees the development of a quality PAS, due to the ability to apply their knowledge and skills to a job and perform it effectively (Aldana-Becerra and Ruiz, 2010; Agencia Nacional de Evaluación de la Calidad y Acreditación [ANECA], 2013; Campos-Izquierdo and Martín-Acero, 2016).

Regarding the least valued professional competences such as leadership and use of new technologies, necessary steps should be taken to promote their application among the professionals involved in the PAS for people with disabilities, because they can directly affect the quality of professional performance. Through the competence of leadership, a professional can effectively motivate, stimulate and influence others (Avolio and Bass, 1999) and therefore better help people with disabilities to achieve their goals. Furthermore, considering the flexibility and adaptation capacity of the new technologies, the competence of their application can play an important role in producing a high level professional performance (Sánchez-López et al., 2014).

It was found that the perception of the importance of professional competences varies among professionals with higher education and those with less training. This should be taken into account, since the highly trained professionals have the ability to effectively apply their knowledge and skills (Aldana-Becerra and Ruiz, 2010; Campos-Izquierdo and Martín-Acero, 2016) and therefore their perception is much more likely to lead to quality professional performance.

The accurate determination of the levels of importance the professionals give to competences prepares the ground for the adequate provision of quality PAS for people with disabilities. Therefore it should be used as a frame of reference for training programs and promoted as transversal axes in the different training programs, thus linking initial training and the acquisition of skills (Campos-Izquierdo and Martín-Acero, 2016). This way a training and competence profile (Jiménez and Hernández-Álvarez, 2013) that guarantees performance professional can be formulated. This aspect is of significant importance, as the different studies show, the PAS professionals working with people with disabilities generally perceive a lack of training and low levels of competence when developing their professional skills (Kelly and Gansneder, 1998; Díaz-Cueto, 2009; Crawford, 2011; Klavina and Kudlácek, 2011; Sanz and Reina, 2012; Jiménez and Hernández-Álvarez, 2013; Castro et al., 2018).

This classification extracted from one’s own professional experience guarantees that professional competencies directly and significantly influence the performance of all PAS job functions with people with disabilities, and can lead to quality PAS and complies with the provisions stated in the Convention on Rights of people with disabilities in 2006.

### Limitations of the Study

This study has some limitations which need to be considered. Perhaps the most important one is the quantitative the nature of this study which does not allow to understand why some PAS professionals working with people with disabilities prefer certain competences over the others. One line of research based on a qualitative approach, focusing on the differences attributed to competencies according to initial training and professional experience. The assessment of professional competences by people with disabilities who access PAS services can also provide a complete vision of the competences (professional-user).

Additionally, the type of disability has not been taken into account in the study. Therefore a possible future line of research would be analysis of the degree of importance given to professional competencies according to the disability (physical, sensory or intellectual). In this way, it could further adjust the professional practice and therefore guarantee the adequacy of the professional to the job in a more effective way.

## Data Availability Statement

The original contributions presented in the study are included in the article/supplementary material, further inquiries can be directed to the corresponding author.

## Ethics Statement

The studies involving human participants were reviewed and approved by the Ethics Commission of the Polytechnic University of Madrid. Written informed consent for participation was not required for this study in accordance with the national legislation and the institutional requirements.

## Author Contributions

MG-C, M-DG-R, and AC-I have carried out an important, direct and intelectual contribution to the work, and approved it for publication.

## Conflict of Interest

The authors declare that the research was conducted in the absence of any commercial or financial relationships that could be construed as a potential conflict of interest.

## Publisher’s Note

All claims expressed in this article are solely those of the authors and do not necessarily represent those of their affiliated organizations, or those of the publisher, the editors and the reviewers. Any product that may be evaluated in this article, or claim that may be made by its manufacturer, is not guaranteed or endorsed by the publisher.
